# Book Review: The Body Keeps the Score: Brain, Mind, and Body in the Healing of Trauma

**DOI:** 10.3389/fpsyg.2021.704974

**Published:** 2021-08-18

**Authors:** Jacqueline Mei Chi Ho, Alex Siu Wing Chan, Ching Yu Luk, Patrick Ming Kuen Tang

**Affiliations:** ^1^School of Nursing, The Hong Kong Polytechnic University, Hong Kong, China; ^2^Department of Applied Social Sciences, The Hong Kong Polytechnic University, Hong Kong, China; ^3^Department of Paediatrics and Adolescent Medicine, Queen Mary Hospital, Hospital Authority, Hong Kong, China; ^4^State Key Laboratory of Translational Oncology, Department of Anatomical and Cellular Pathology, The Chinese University of Hong Kong, Hong Kong, China

**Keywords:** posttraumatic stress disorder, physical well-being, psychological well-being, health care, quality of life

Posttraumatic stress disorder (PTSD) is defined as a psychiatric disorder in an individual who has experienced or witnessed a devastatingly traumatic event (Bufka et al., 2020). These patients persistently experience overwhelming stress responses beyond the traumatic period. Fear and stress are triggered predominantly in response to a dangerous event followed by a series of bodily changes, including increased blood pressure, heart rate, and breathing due to the release of adrenaline. This biochemical reaction is termed as the “fight-or-flight” mechanism, which temporarily protects people against danger (Kozlowska et al., 2015). It has been well-recognized that PTSD significantly impacts social functioning, physical well-being, and occupational ability (Watkins et al., 2018).

Bessel revisits his clinical experience and reconfirms the impact of PTSD on his patients. People with PTSD experience various symptoms, including personality changes, depression, social disconnection, poor sleep hygiene, flashbacks, and nightmares. Facing recurrent episodes of disturbing symptoms, PTSD patients are prone to develop risky behaviors, including alcoholism, substance abuse, or self-injury. PTSD has many negative impacts on patients' quality of life (QoL), and poses a potential health burden to society if no prompt assessment and support is guaranteed (Lewis et al., 2019).

In his work “The body keeps the score,” Bessel highlights that traumatic stress is at the root of neuroscience. Traumatic stress is associated with functional and chemical changes in the emotional part of the brain—the limbic area and brain stem. Knowing the functions of the amygdala, hippocampus, and prefrontal cortex, as the primary stress responders in the brain, can provide a new therapeutic direction for PTSD management. The hyperactive status of the amygdala triggers the release of stress hormones (Badura-Brack et al., 2018) and impairs the functioning of the hippocampus, causing traumatic memories to remain vivid. In addition, the deactivation of the prefrontal cortex function and the failure to maintain a balanced stress hormone system, causes panic, agitation, and hypervigilance responses in PTSD patients (Koenigs and Grafman, 2009). This hyperactive aroused emotional status can be evidenced by hyperactive brain waves over the fear center of the right temporal lobe of the brain, with suppression of electric activity over the frontal area.

Pharmacotherapy is the first-line of treatment for PTSD. Antipsychotics, anticonvulsants, and tranquilizers have been widely used to improve the QoL of PTSD patients over the past few decades. As some patients developed morbid obesity and diabetes from the medication as well as experienced drug overdoses, it alarmed the book author and pushed him to consider a much safer and natural approach to assist PTSD patients in dealing with their symptoms and responses using a self-regulation strategy. Bessel further suggests that medication cannot 'cure' trauma; it can only mediate the disruptive behavior of the sufferers.

This revolutionary treatment was enlightened by one of Bessel's patients, who could not get rid of his traumatic memory as a minister for many years after returning home from Vietnam. This memory was subconsciously imprinted in his life. Bessel continued working with this patient and explored that yoga can help him regain his sense of control and bodily pleasure. A subsequent experimental study showed that mindfulness yoga significantly reduced PTSD symptomatology and restore the homeostasis of the autonomic nervous system. With the aid of the mindfulness approach, we can raise awareness of bodily sensations, which can improve control over the flow of emotions by decreasing activity over the amygdala. Hence, individuals can have a reasonable degree of control over themselves, both physically and psychologically (Streeter et al., 2012).

To restore the emotional part of the brain and repair the limbic system, Bessel explains various psychotherapies related to the brain, mind, and body of PTSD patients. He emphasized on the human body as the means of communicating with oneself and others. Expressive therapies, through language, art, music, and dance, can motivate people orientate themselves and find their own identity and a meaningful purpose in life (Baker et al., 2018). Most importantly, the reconnection of attachment bonding with family and friends can help individuals feel secure in fighting against the threat (Chan et al., 2021a,b).

“The body keeps the score” attempts to address how PTSD patients experience trauma over the years following a traumatic exposure. From the stories of different victims introduced by the author, Bessel used both scientific and philosophical approaches to explain the complex neurobiology and connection of the human brain-mind-body, and provided useful guides for specialists and the public. An abstract or summary of each chapter can aid readers in capturing the essence of the message. As various approaches that can help people with PTSD are suggested by the author, the book would be more comprehensive if further empirical findings are provided to demonstrate their effectiveness and how readers can integrate them into practice.

## Author Contributions

JH is mainly writing this book review. CL, AC, and PT gave comment and suggestion in this manuscript. All authors contributed to the article and approved the submitted version.

## Conflict of Interest

The authors declare that the research was conducted in the absence of any commercial or financial relationships that could be construed as a potential conflict of interest.

## Publisher's Note

All claims expressed in this article are solely those of the authors and do not necessarily represent those of their affiliated organizations, or those of the publisher, the editors and the reviewers. Any product that may be evaluated in this article, or claim that may be made by its manufacturer, is not guaranteed or endorsed by the publisher.
